# Significant improvement in Mn_2_O_3_ transition metal oxide electrical conductivity via high pressure

**DOI:** 10.1038/srep44078

**Published:** 2017-03-09

**Authors:** Fang Hong, Binbin Yue, Naohisa Hirao, Zhenxian Liu, Bin Chen

**Affiliations:** 1 Center for High Pressure Science and Technology Advanced Research, 1690 Cailun Rd. Pudong, Shanghai 201203, P.R. China; 2 The Advanced Light Source, Lawrence Berkeley National Laboratory, 1 Cyclotron Rd, Berkeley, CA 94720, USA; 3 SPring-8/JASRI, 1-1-1 Kouto, Sayo-cho, Sayo-gun, Hyogo 679-5198, Japan; 4 Geophysical Laboratory, Carnegie Institution of Washington, Washington, DC 20015, USA

## Abstract

Highly efficient energy storage is in high demand for next-generation clean energy applications. As a promising energy storage material, the application of Mn_2_O_3_ is limited due to its poor electrical conductivity. Here, high-pressure techniques enhanced the electrical conductivity of Mn_2_O_3_ significantly. *In situ* synchrotron micro X-Ray diffraction, Raman spectroscopy and resistivity measurement revealed that resistivity decreased with pressure and dramatically dropped near the phase transition. At the highest pressure, resistivity reduced by five orders of magnitude and the sample showed metal-like behavior. More importantly, resistivity remained much lower than its original value, even when the pressure was fully released. This work provides a new method to enhance the electronic properties of Mn_2_O_3_ using high-pressure treatment, benefiting its applications in energy-related fields.

Transition metal oxides have unique properties that are capable of developing new, functional and smart materials. In particular, manganese oxide has been a key focus due to its outstanding structural diversity and novel physicochemical properties^1^,^2^,^3^,^4^. It also has the advantage of being earth abundant, non-toxic and cost effective, attracting enormous attention for a wide variety of energy and environmental applications, such as chemical catalysts, magnetic devices, and energy conversion and storage^5^,^6^,^7^,^8^,^9^,^10^. Mn_2_O_3_ is a semiconductor but it can be employed as a high-performance anode material in Lithium-ion batteries (LIBs), with a high theoretical capacity of 1018 mAh/g at a low operating voltage (charge voltage at 1.2 V and discharge voltage at 0.5 V)^11^. Furthermore, its one electron transfer caused by electrochemical redox activity via the Mn^3+^ to Mn^4+^ transition is significant for its electrode reactions in electrochemical capacitors.

Electrode materials for batteries and supercapacitors must have good electrical conductivity to achieve high electrochemical performance^12^,^13^. Poor electrical conductivity may limit the solid-state diffusion rate of the electrons and/or ions, consequently resulting in reduced power and energy density. In this regard, the poor electrical conductivity of Mn_2_O_3_ is a major drawback. Traditionally, the electrical conductivity of transition metal oxides can be enhanced by forming nanocomposite materials with highly conductive carbon or graphene. Alternatively, cationic doping achieves a similar effect^14^,^15^. In addition, various nanostructures like nanorods, nanospheres, and mesoporous Mn_2_O_3_ have been prepared and demonstrate a clear improvement in the specific capacitance^16^,^17^,^18^. These methods have undoubtedly boosted the development of transition metal oxide-based energy storage. However, sometimes these methods have additional problems, such as low thermal stability and an increased manufacturing cost. Therefore, improving the intrinsic electrical conductivity of transition metal oxides for high-performance energy storage is imperative. Recent works have shown pressured-induced electron transport enhancement of Nb-doped TiO_2_ nanoparticles and single-crystal Ta_2_O_5_ nanowires^19^,^20^ and there are further reports of pressure-induced superconductivities^21^,^22^. Therefore, pressure may be a powerful tool to modify the electrical conductivity of transition metal oxides.

Here, we tentatively studied the electrical properties of the promising transition metal oxide, Mn_2_O_3_, using high-pressure to reveal the relationship between its electrical properties and structure. First, we investigated the electrical properties *in situ* inside a diamond anvil cell by resistance measurements at room temperature and pressures up to 43 GPa. The electrical conductivity enhanced by five orders of magnitude at the highest pressure. More importantly, conductivity after high-pressure treatment was more than ten times higher than the original level before treatment at ambient conditions. To reveal the mechanism of this enhancement behavior, we examined the structural evolution of Mn_2_O_3_ under high pressure using *in situ* synchrotron x-ray diffraction (XRD) and Raman spectroscopy at room temperature; a phase transition began around 18.5 GPa and 15 GPa, respectively. We also examined the electronic structure by infrared spectroscopy and proved that Mn_2_O_3_ behaves like a metal rather than a semiconductor under high pressure. This work provides an efficient way to improve the electrical conductivity of manganese oxides, benefiting their applications as electrode materials for high-performance batteries and supercapacitors.

## Results and Discussion

### The electrical measurement of Mn_2_O_3_ under high pressure

**Figure 1:**
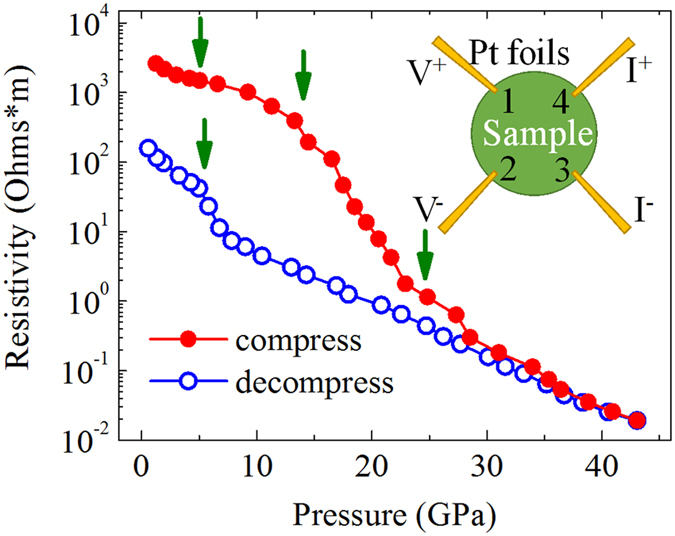
The pressure dependent resistivity of Mn_2_O_3_ during compression and decompression. Insert: the scheme of electrical measurements in the diamond anvil cell.

Figure 1 displays the pressure dependent electrical resistivity of Mn_2_O_3_ during both compression and decompression. During compression, resistivity shows a clear decreasing trend with increasing pressure and a huge resistivity drop occurs. At the highest pressure of ~43 GPa, the resistivity is only on the scale of ~10^−2^ Ohms*m compared to ~10^3^ Ohms*m near ambient conditions. A sharp resistivity change from ~15 GPa to ~25 GPa indicates an electronic transition. In addition, a minor kink near 5 GPa may also suggest an electronic transition. This transition is much clearer when the resistivity-pressure curve is plotted on a linear scale, as shown in Figure S1 in the Supplementary information. During decompression, the pressure dependent resistivity trend is the opposite. It increases with pressure release and a kink appears near 5 GPa, which may be related to an inverse electronic transition. When pressure is fully released, resistivity remains one order of magnitude lower than it was before compression.

Interestingly, the relationship between resistivity and pressure can be well described by a logarithmic function for each phase. To exclude the grain boundary effect and mixed phases, we only fitted the decompression data with logarithmic functions. The fitting details are provided in Figure S2 in the Supplementary information. The pressure dependent resistivity can be expressed by the following relationship:

For the high-pressure range:


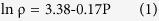


For the low-pressure range:


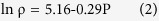


where ρ is the resistivity and P is the pressure. Similar logarithmic behavior of pressure dependent resistivity has also been found in VO_2_, MoSe_2_, and GeSb_2_Te_4_^23^,^24^,^25^.

### The crystal structure investigated by synchrotron x-ray diffraction and Raman spectroscopy under high pressure

**Figure 2:**
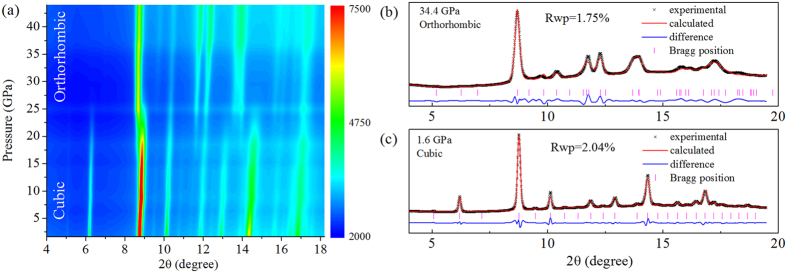
The X-ray diffraction results of Mn_2_O_3_ up to ~44 GPa (wavelength is 0.4141 Å) during compression. (**a**) Two-dimensional X-ray diffraction patterns. (**b**,**c**) The representative refinement patterns at 34.4 GPa and 1.6 Gpa, respectively. A structural phase transition is observed near 18 GPa and finishes near 26.5 GPa. The refinement merit is indicated by the Rwp values, which are very small.

To understand the mechanism of the enhanced electrical conductivity, we examined the Mn_2_O_3_ structural evolution under high pressure. Figure 2 demonstrates the X-ray diffraction patterns at various pressures during compression. At low pressure, the structure of Mn_2_O_3_ can be well assigned to a cubic phase with the *Ia-3* space group^26^. New diffraction peaks appear clearly from 18.5 GPa, suggesting that a phase transition has already occurred. The new phase mixes with the cubic phase from 18.5 GPa to 26.5 GPa before the cubic phase is completely suppressed above 26.5 GPa. This high-pressure phase can be assigned to an orthorhombic structure with the space group *Cmcm*^26^. Figure 2(b,c) shows representative refinement patterns of Mn_2_O_3_ collected at 34.4 GPa and 1.6 GPa, corresponding to the high-pressure orthorhombic phase and the low-pressure cubic phase, respectively. The refinement merit is indicated by the Rwp values, which are quite small at only 1.75% and 2.04%. The structural details of these two pressure points are listed in Table S1 in the Supplementary information.

**Figure 3:**
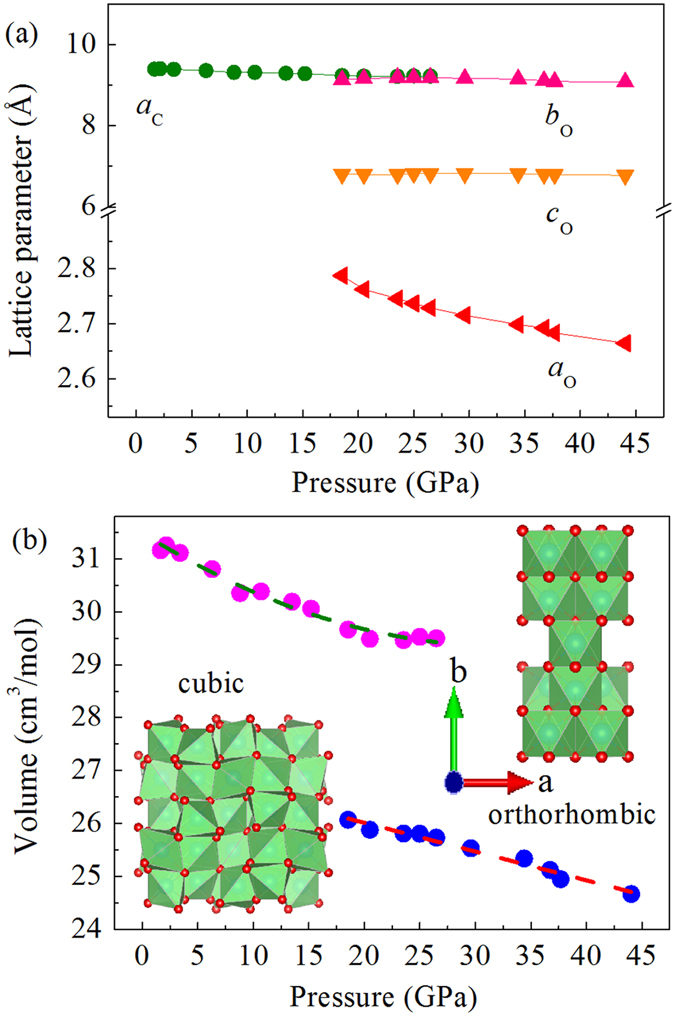
Refinement results of Mn_2_O_3_ structural information during compression. (**a**) The pressure dependent lattice parameters. (**b**) Volume evolution under high pressure (inset: atomic structures of cubic and orthorhombic phases). There is a large volume collapse at the phase transition pressure. At ~18.5 GPa, the volume difference of the two phases reaches ~12%.

The pressure dependent structural information is presented in Fig. 3. Figure 3(a) displays the pressure dependent lattice parameters. For the cubic phase, the lattice parameter decreases monotonously with pressure. For the orthorhombic phase, the *a* axis shows a clear declining trend with pressure, while the c axis shows little change and the b axis decreases slowly above the final phase transition pressure of 26.5 GPa. Figure 3(b) demonstrates the volume evolution with pressure; the large change in the atomic structure causes a significant volume change near the phase transition pressure. The unit volume of the orthorhombic structure at 18.5 GPa is only ~87.5% of the cubic structure. In the high-pressure orthorhombic structure, oxygen atoms in the *ab*-plane show a highly ordered arrangement and the MnO_6_ units are less distorted compared to those in the cubic structure, as demonstrated by the insets in Fig. 3(b). Large pressure-induced volume changes in transition metal compounds are usually related to an electronic structural change of the transition metal ions (such as Fe, Co, and Mn), which can undergo a spin state transition from a high/intermediate spin state to a low spin state^27^,^28^,^29^. The spin state transition of MnS_2_ has a volume collapse as large as 22% from the low-pressure cubic structure to a high-pressure monoclinic structure near 18 GPa. Recently, a spin state transition was also observed in MnS and MnSe with a large volume collapse^30^. Here, the large volume collapse in our Mn_2_O_3_ may originate from a spin state transition too but further study is required to clarify this assumption.

We also investigated the Mn_2_O_3_ structural evolution by Raman spectroscopy during compression. The results are provided in Figure S3 in the Supplementary information. A structural phase transition occurred near 15 GPa, indicated by two new, strong vibration modes (~550 and ~650 cm^−1^ at 20 GPa). Two extra modes located at ~150 and ~200 cm^−1^ became more pronounced when the pressure reached ~18 GPa, suggesting an overall phase transition occurred. This is well consistent with the XRD results and no further change was observed in the current pressure limit. The Raman spectra results confirmed that the structural phase transition started ~15 GPa, a little bit lower than the XRD result determination. The phase transition also matched the resistivity measurement.

We studied the compressibility of the Mn_2_O_3_ low-pressure (cubic) and high-pressure (orthorhombic) structures using second-order Birch-Murnaghan equation of state (EOS) analysis^31^,^32^. The fitting results are presented in Figure S4 in the Supplementary information. For the cubic phase, V_0_ is 34.43 cm^3^/mol and the bulk modulus B_0_ is 286.4 ± 16.6 GPa, using a fixed first-order pressure derivative of the bulk modulus B′ = 4. For the orthorhombic phase, V_0_ is 27.5 cm^3^/mol and the bulk modulus B_0_ is 331.09 ± 28.3 GPa, using a fixed first-order pressure derivative of the bulk modulus B′ = 4. Hence, the high-pressure phase is a little more difficult to compress than the low-pressure phase.

**Figure 4:**
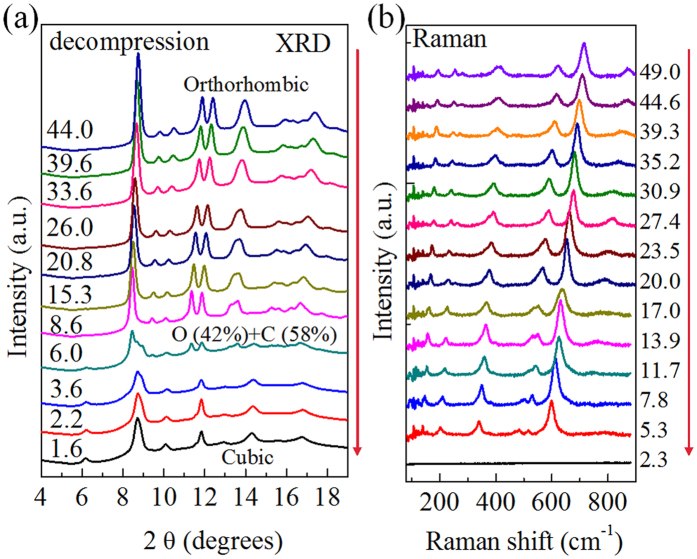
Structural investigation during decompression. (**a**) Pressure dependent X-ray diffraction patterns. (**b**) Pressure dependent Raman spectra. Pressure unit: GPa.

To reveal the mechanism of the different electrical resistivity behavior under compression and decompression, XRD and Raman spectra were also collected during decompression, as shown in Fig. 4(aand b), respectively. No phase transition occurred when the pressure was released to 8.6 GPa, as confirmed by our XRD results. However, a clear difference was visible near 6.0 GPa, where an extra diffraction peak appeared. Subsequently, the low-pressure phase dominated and we assigned this phase to the previous cubic phase, though the peaks broadened and the intensity of some peaks was lower, compared with those during compression. The broadening and intensity changes were due mainly to the reduced crystal size and disorders induced by pressure. The Raman spectra gave similar results. No clear change presented until pressure reduced to 5.3 GPa. After that, the intensity of the Raman signal almost disappeared and no peaks were identified. This was consistent with the cubic phase, where the Raman signal was much weaker than in the orthorhombic phase. The disorders and smaller crystal size also affect the Raman signal in the low-pressure range, which made the weak Raman signal much more difficult to collect. These results match well with previous reports of high-pressure phase stability until 4.5 GPa during decompression^33^,^34^. More importantly, the structural change during decompression and the resistance measurement results are also consistent, as displayed in Fig. 1. Hence, the electronic transition near 5 GPa originates from a reversible structural phase transition. Similar results have been reported on other materials^20^. The microstructural change after high-pressure treatment should be responsible for the enhanced conductivity behavior. Pressure can drive lattice distortion and even slip^35^,^36^, during which defects appear, breaking the original locally neutral charge distribution and aiding electron transfer.

### Infrared spectroscopy study on Mn_2_O_3_ under high pressure

**Figure 5:**
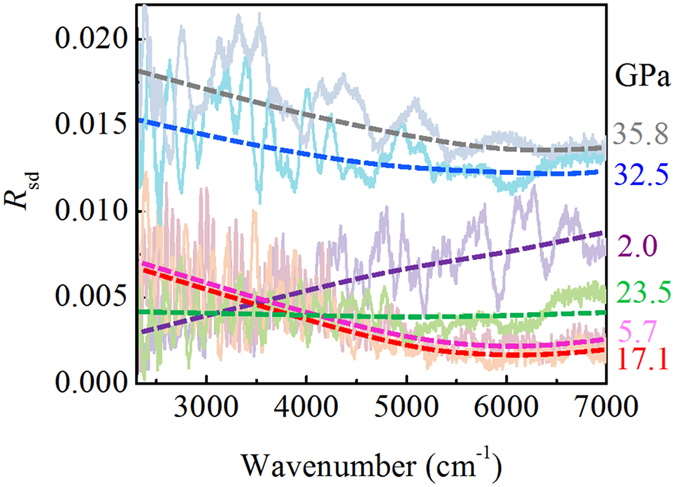
The IR reflectance spectroscopy of Mn_2_O_3_ under high pressure. Some changes at certain pressures (5.7 GPa, 23.5 GPa, 32.5 GPa) indicate electronic structural changes.

The electronic behavior of Mn_2_O_3_ under high pressure was investigated by synchrotron-based infrared spectroscopy (IR) to reveal the electronic structural change. Figure 5 presents the reflectivity spectra at some typical pressures. As the sample we used in the IR measurement was a fine powder, the reflectivity signal was noisy and relatively low due to strong scattering on the coarse surface. However, we still observed a clear trend under high pressure, which helps us understand its electronic behavior. An average dot plot for each spectrum guides the eyes. We expected the spectrum at 2.0 GPa to be similar to that at ambient conditions. It shows typical reflective behavior from a semiconductor or insulator, where reflectance is lower in a low wave number (low-energy) region than in a high wave number (high-energy) region. No obvious change occurred when the pressure increased a little from 2.0 GPa to 3.7 GPa (shown in Figure S5(a) in the Supplementary information). The trend suddenly changed at 5.7 GPa, which is characteristic of metal-like behavior as reflectance is higher in the low wave number region than in the high wave number region. This is also consistent with the resistance measurement result during compression in Fig. 1, where a kink occurs near 5 GPa. This change suggests that a previously unreported electronic phase transition occurred near 5 GPa. Above 5.7 GPa, the reflectance slightly increased with pressure in the long wavenumber range (shown in Supplementary information Figure S5(b)), suggesting better conductivity and metal-like behavior. This trend changed when the pressure reached 17.1 GPa, near the structural phase transition. The reflectance decreased at 17.1 GPa to even lower than at 5.7 GPa. Another change occurred between 23.5 GPa and 26.7 GPa where reflectance strengthened due to the completion of the structural phase transition. Figure 5 shows stronger reflection at 32.5 GPa or higher pressure, and this trend follows a metal-like behavior. To briefly summarize the observed electronic behavior; the sample underwent an electronic phase transition near 5.7 GPa with no structural change and a structural phase transition induced electronic structure change near 17.1 GPa. The phase transition concluded between 23.5 and 26.7 GPa. These IR results agree with the resistance measurements and structural analysis.

## Conclusion

In summary, we systematically investigated the electronic and structural properties of Mn_2_O_3_ under high pressure using resistivity measurements, X-ray diffraction, Raman spectroscopy, and infrared spectroscopy. A cubic-orthorhombic phase transition began near 15.1 GPa. The resistivity dropped sharply near this transition and the sample showed some metal-like features at higher pressure. Clearly, high pressure strongly enhanced electrical conductivity. The quenched sample maintained better electrical conductivity than its original value. Our work helps solve the problem of poor Mn_2_O_3_ electrical conductivity without the addition of another element, allowing wider applications in the energy storage field.

## Methods

### High-pressure XRD, Raman, and IR study at room temperature

The α-Mn_2_O_3_ (99.99% trace metals basis) was purchased from Sigma-Aldrich. *In situ* Raman spectra and X-ray diffraction patterns under various pressures were collected from the α-Mn_2_O_3_ sample, which was loaded into a Mao-type symmetric diamond anvil cell (DAC) with a diamond culet of 300 μm^37^,^38^. Silicone oil was used as a pressure medium. Reports suggest that silicone oil hydrostaticity is as good as a 4:1 methanol:ethanol mixture at low pressures to ~20 GPa and behaves like argon above 30 GPa^39^,^40^. The Raman spectra were collected using a micro-confocal Renishaw Raman system with a 532 nm green laser^38^. A stainless steel gasket was used and a 100 μm sample hole was drilled with a laser drilling system. The pressure was monitored by the Ruby R1-R2 line shift. The *in situ* synchrotron micro X-ray diffraction experiment was carried out at Beamline 10XU in Spring-8 and the incident X-ray wavelength was 0.4142 Å. The patterns were collected using a Perkin Elmer digital X-ray flat panel detector (FPD, XRD0822 CP23; 1024 × 1024 pixels; 0.2 mm pixel pitch). Infrared spectra were collected *in situ* inside the DAC using IR diamonds at Beamline 1.4.4 in the Advanced Light Source at Lawrence Berkeley National Laboratory, using KBr as the pressure medium.

### High-pressure electrical resistivity measurements at room temperature

*In situ* high-pressure electrical resistance measurements were conducted with a four-probe resistance test system in a DAC at pressures up to 43.09 GPa. The mixture of boron nitride (BN) and epoxy was loaded between the sample and the gasket to provide an electrical insulation layer. The other parts of the gasket were covered with insulating glue to avoid contact between the conductive leads and metal gasket. The sample was loaded into the BN hole without a pressure medium. Four platinum foils were arranged to contact the sample in the chamber. A Keithley 6221 current source and 2182A nano-voltmeter were used as the current supply and voltmeter, respectively. The resistance was determined by the Van de Pauw method, similar to that described in reference^41^.

## Additional Information

**How to cite this article:** Hong, F. *et al*. Significant improvement in Mn_2_O_3_ transition metal oxide electrical conductivity via high pressure. *Sci. Rep.* **7**, 44078; doi: 10.1038/srep44078 (2017).

**Publisher's note:** Springer Nature remains neutral with regard to jurisdictional claims in published maps and institutional affiliations.

## Supplementary Material

Supplementary Information
