# Supplementary material for: Significant improvement in Mn2O3 transition metal oxide electrical conductivity via high pressure
Source: Sci Rep. 2017 Mar 9;7:44078. doi: 10.1038/srep44078 (PMC5343433; doi:10.1038/srep44078)
Supplement: Supplementary Information [file srep44078-s1.pdf]

Supporting Information for

Significant improvement in Mn_2O_3 transition metal oxide electrical conductivity via high pressure

Fang Hong[1, 2], Binbin Yue[1, 2*], Naohisa Hirao[3], Zhenxian Liu[4], Bin Chen[1*]

[1]*Center for High Pressure Science and Technology Advanced Research, 1690 Cailun Rd. Pudong, Shanghai 201203, P.R. China*

[2]*The Advanced Light Source, Lawrence Berkeley National Laboratory, 1 Cyclotron Rd, Berkeley, CA 94720, USA*

[3]*SPring-8 / JASRI, 1-1-1 Kouto, Sayo-cho, Sayo-gun, Hyogo 679-5198, Japan*

[4]*Geophysical Laboratory, Carnegie Institution of Washington, Washington, DC 20015, USA*

*email: byue@lbl.gov; chenbin@hpstar.ac.cn

Content

1. The linear plot of resistivity-pressure curve……………………………….…..…2

   Figure S1……………………………………………………………….…...2

2. The fitting of resistance-pressure plots……………………………….........3

   Figure S2……………………………………………………………….…...3

3. Structural information for low pressure and high pressure phases…………....4

   Table S1………………………………………………………….........4

4. Raman spectra collected during compression process………………….........5

   Figure S3……………………………………………………………….…...5

5. EOS analysis of low pressure and high pressure phases……………………..…..7

   Figure S4…………………………………………………………...…....7

6. The electronic transition near 5 GPa and 15GPa confirmed by infrared spectroscopy……………………………………………………………..…8

   Figure S5………………………………………………………..…..8

1. The linear plot of resistivity-pressure curve

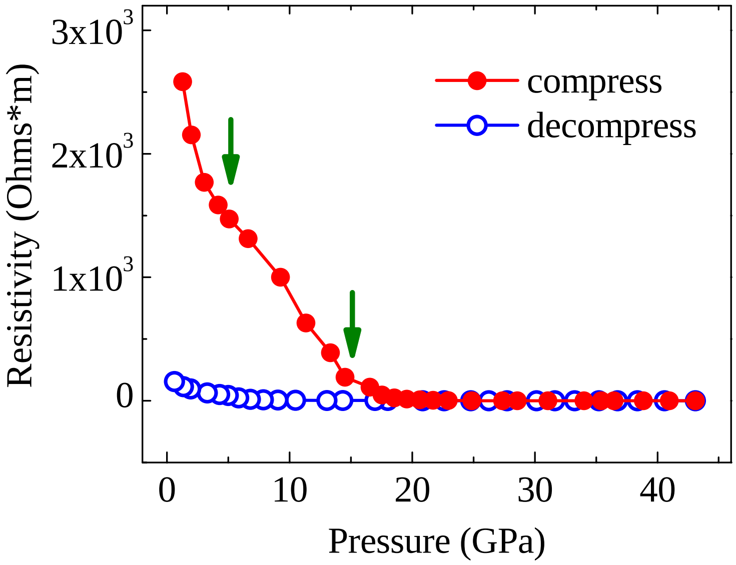

Figure S1 The pressure dependent resistivity during compression and decompression processes

There are two possible electronic transitions confirmed by the electrical resistivity measurement, as indicated by the arrows. The first one is near 5 GPa and the second one locates near 15 GPa. An extra transition can be observed near 25 GPa in Figure 1 which is plotted in logarithmic scale displayed in the main text.

2. The fitting of resistance-pressure plots

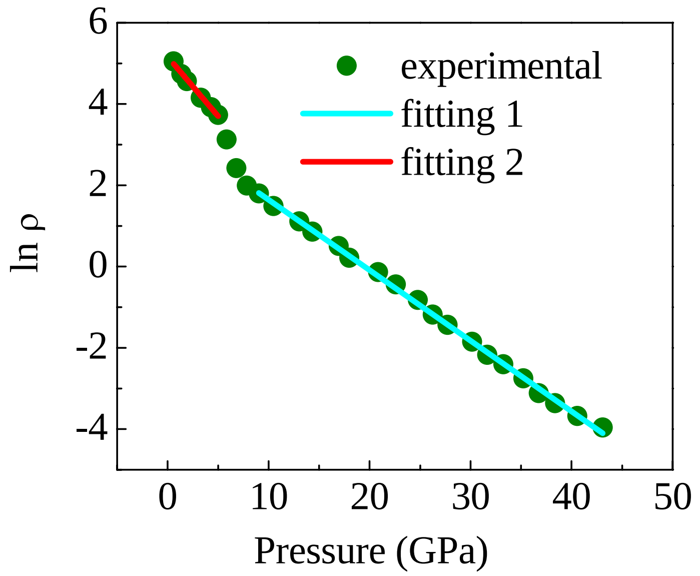

Figure S2 The fitting of ln(resistivity)-pressure plot

To extract the relation between resistivity and pressure, a linear fitting was done based on the ln(resistivity)-Pressure curve by using Origin software. A good fit result is obtained and the fitting merit is described by the value of "Residual Sum of Squares" AND "Adj. R-Square". Cleary, the residual is very small and R-Square value is close to 100%, which suggests a nice fitting.

At high pressure, the parameter is presented as following, which corresponds to fitting 1.

Equation		y = a + b*x	
Residual Sum of Squares		0.08759	
Adj. R-Square		0.99845	
		Value	Standard Error
Resistivity	Intercept	3.38527	0.04499
	Slope	-0.17371	0.00161

At low pressure, the parameter is presented as following, which corresponds to fitting 2.

Equation		y = a + b*x	
Residual Sum of Squares		0.00927	
Adj. R-Square		0.99104	
		Value	Standard Error
Resistivity	Intercept	5.16376	0.03943
	Slope	-0.2927	0.01244

3. Structural information for low pressure and high pressure phases

Table S1 The detailed structural information of low pressure cubic and high pressure orthorhombic phase at 1.6 GPa and 34.4 GPa, respectively

**Low pressure phase**		**Cubic (*Ia-3*)**	**Z=16**	**a= 9.391(2)**	**1.6 GPa**
	site	x	y	z	occupancy
Mn1	8b	0.25	0.25	0.25	1
Mn2	24d	-0.0337(2)	0	0.25	1
O	48e	0.3808(2)	0.1613(8)	0.3982(1)	1

**High pressure phase**		**Orthorhombic (*Cmcm*)**	**Z=1**		**34.4 GPa**
		**a**	**b**	**c**	
		2.690(5)	9.152(3)	6.815(7)	
	site	x	y	z	occupancy
Mn1	4a	0	0	0	1
Mn2	4c	0	0.2574 (5)	0.25	1
O1	4c	0	0.8799 (8)	0.25	1
O2	8f	0	0.6082 (0)	0.4217 (8)	1

4. Raman spectra collected during compression process

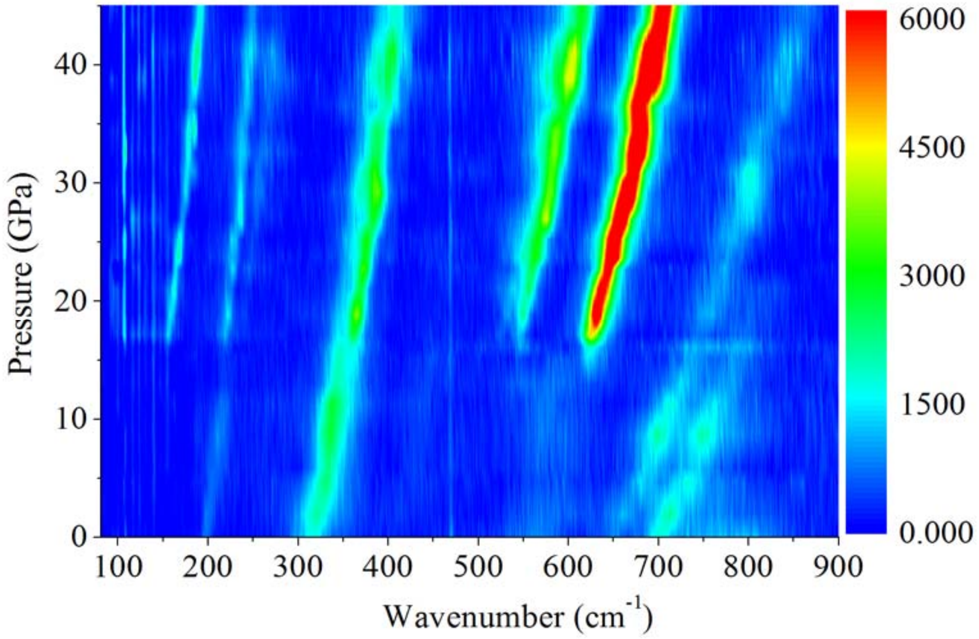

Figure S3 Raman spectra of Mn_2O_3 collected during the compression process. A phase transition is observed near 15 GPa, where extra peaks appear. No further structural change is found in current work.

To define the phase transition pressure more accurately, we also employed Raman spectroscopy, which is much more sensitive to a change in the local structure. The Raman signal of the cubic phase was very weak and every spectrum took more than 30 minutes. In spite of the weak signal, we still obtained the characteristic vibration modes. There are a few reports related to the Raman vibration modes of Mn_2O_3 and their Raman spectra look quite different.[1-3] Mn_2O_3 is a known semiconductor with a band gap of ~3.27 eV and our Mn_2O_3 sample showed a natural black color, suggesting its strong visible light absorption ability. Therefore, there is a possibility that the Mn_2O_3 structure may change under strong light radiation, during which different Raman Spectra can be obtained. We then studied the Raman spectra of Mn_2O_3 excited by different powers. A strong signal but totally different spectrum was obtained when excited by a laser with high power. This means that only the weak signal was real from Mn_2O_3 itself.

Figure S3 displays the Raman spectra collected *in situ* under pressures up to ~45 GPa. As mentioned above, the Raman signal is weak at ambient conditions and the sample can be easily damaged by the high power laser. As pressure increases, the signal intensifies; see the spectrum at 8.6 GPa, for example. All modes show blue shift with pressure, which is due to the shorter bond distance under high pressure. A new mode near 620 cm^{-1} is found when the pressure reaches 15.1 GPa, suggesting the onset of a phase transition, which is earlier than the results from the XRD analysis. When the pressure reaches 18.8 GPa, two new, extra Raman modes are observed and the intensity of the mode near 620 cm^{-1} suddenly increases. No further big changes occur above 18.8 GPa. Nine Raman modes were observed for this high pressure phase as reported by Shim *et al.*.[4] This is very close to the phase transition pressure of 18.5 GPa, as determined by our XRD results. Based on this analysis, we conclude that the phase transition starts near 15.1 GPa with local structure changes revealed by the Raman spectroscopy, and the overall structure change starts near 18.5 GPa, as detected by XRD.

5. The EOS analysis of the low pressure and high pressure phases

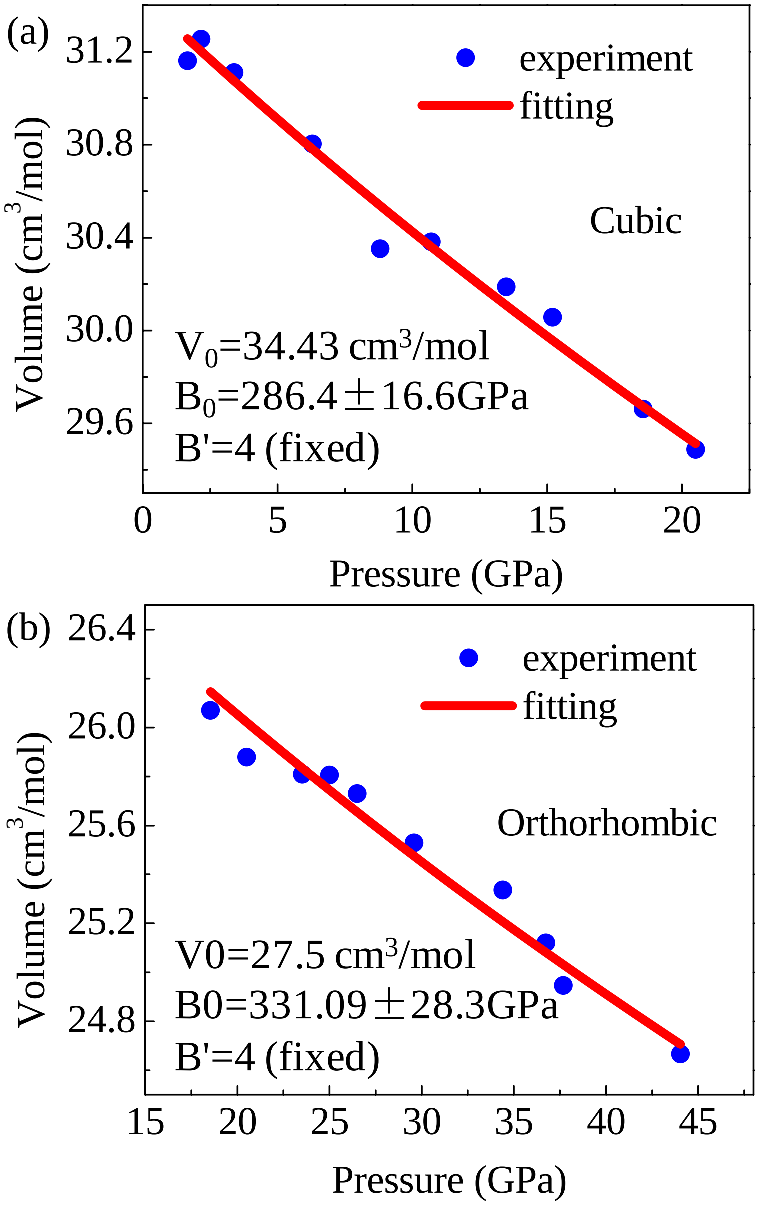

Figure S4 The EOS analysis. (a) The low pressure cubic phase; (b) the high pressure orthorhombic phase.

6. The electronic transition near 5 GPa confirmed by infrared spectroscopy

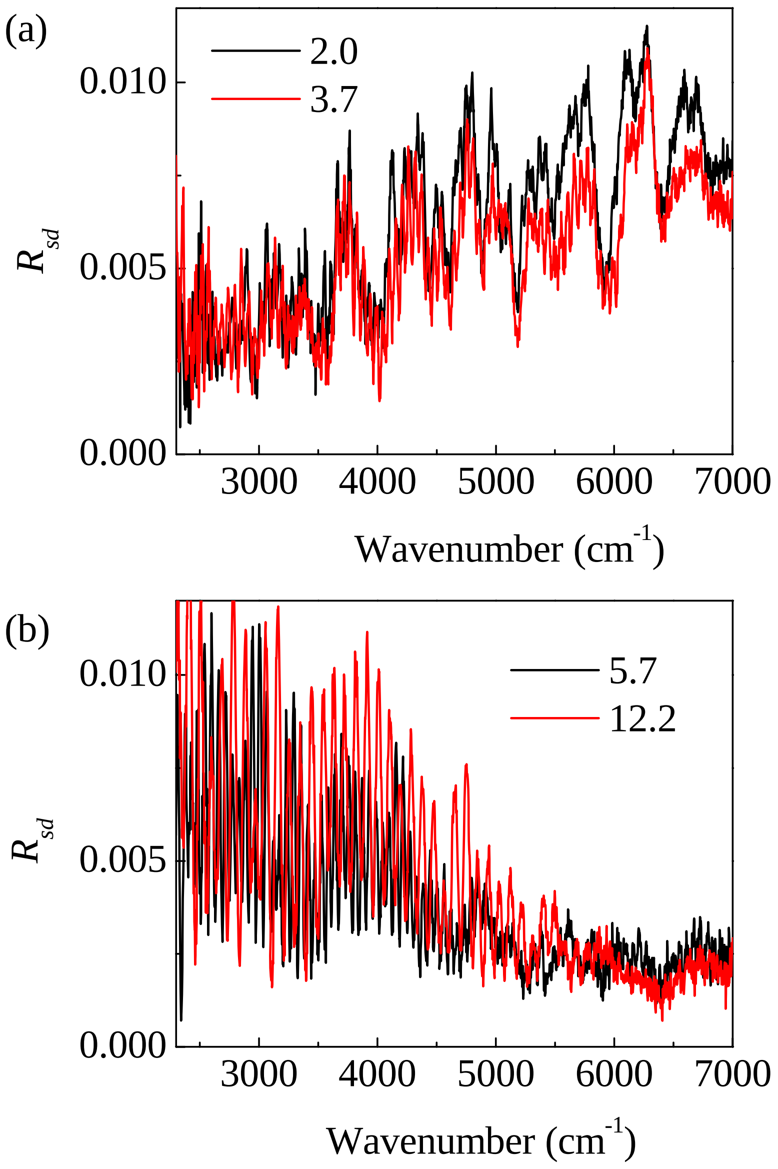

Figure S5 The infrared spectroscopy reveals the appearance of an electronic transition near 5 GPa.

In **Figure S5 (a)**, the spectra collected at 2.0 and 3.7 GPa are almost identical, suggesting that they are in the same state. When pressure reaches 5.7 GPa, the spectrum is totally different from those at 2.0 and 3.7 GPa, as shown in **Figure S5(b)**. Further increase on pressure to 12.2 GPa, the spectrum changes slightly and shows the similar trend with that at 5.7 GPa. This confirms that there is an electronic transition near 5 GPa while the structure doesn't change.

[1] Y. Luo, Y.-Q. Deng, W. Mao, X.-J. Yang, K. Zhu, J. Xu, Y.-F. Han, *J. Phys. Chem. C* **2012**, *116*, 20975-20981.

[2] K. Ramesh, L. Chen, F. Chen, Y. Liu, Z. Wang, Y.-F. Han, *Catal. Today* **2008**, *131*, 477-482.

[3] X. Niu, H. Wei, K. Tang, W. Liu, G. Zhao, Y. Yang, *RSC Advances* **2015**, *5*, 66271-66277.

[4] S. H. Shim, D. LaBounty, T. S. Duffy, *Phys Chem Minerals* **2011**, *38*, 685-691.
